# Supplementary material for: Antibiotic perceptions, adherence, and disposal practices among parents of pediatric patients
Source: PLoS One. 2023 Feb 9;18(2):e0281660. doi: 10.1371/journal.pone.0281660 (PMC9910628; doi:10.1371/journal.pone.0281660)
Supplement: S1 Fig — (see attached REDCap tool). (PDF) [file pone.0281660.s001.pdf]

Parental Perceptions about Antibiotics Survey

Enrollment Information:

1) Did caregiver sign consent form?
   ◯ Yes
   ◯ No
   (Write record ID number on consent form.)

2) Today's Date ______________________

3) Date of follow-up visit: ______________________

4) Time of follow-up visit: ______________________

5) Address of follow-up visit: ______________________

6) Was NP specimen collected?
   ◯ Yes
   ◯ No

7) Pre-antibiotic NP specimen number: ______________________

Part I: Greetings and patient status check

Good morning/afternoon, my name is _________ and my colleague here is ____________. We work in research at Vanderbilt. Thank you for participating in our study. We understand (name of child) was sick recently. We hope he/she is feeling better since we last met you at (name of clinic).

I am going to ask you some questions. Please answer them to the best of your ability. There are no right or wrong answers to these questions, and your answers will help us better understand what you think about certain medications. Your answers will be recorded, but they will remain anonymous so that it will not be possible to identify who answered our questions.

8) How is (name of child) doing in terms of his recent infection since starting [his/her] antibiotic medication? Would you say [he/she] is _________?

- ◯ Greatly improved
- ◯ Slightly improved
- ◯ About the same
- ◯ Somewhat worse
- ◯ A lot worse

(Read the following answer choices. If the caregiver needs clarification, prompt him or her to focus on how the child is doing today compared to his/her doctor visit last week.)

Part II: Parental knowledge/attitudes towards antibiotics

What medications were given to your child last week at (name of clinic)? [Pause here and allow caregiver to respond. No need to record answer.] Can you please bring them to show us? [Wait for caregiver to bring medicines. Point towards antibiotic.] As you mentioned, your child was prescribed an antibiotic called (name of antibiotic). We are now going to talk about antibiotics for the remainder of this conversation.

9) When you hear the word antibiotic, what comes to mind?

____
(Note that caregivers can provide an answer or indicate that they are not sure or that they do not know. All those answers are valid and should be recorded as such in the free text box.)

10) Why do you think your doctor prescribed the antibiotic for your child?

____

11) How do you think the antibiotic works?

____

12) Do you think the antibiotic helped your child get over his/her illness?

◯ Yes
◯ No
◯ I don't know

13) How do you think the antibiotic helped your child?

____

Several medications have side effects. The next few questions are regarding your general thoughts on the side effects of antibiotics. Examples of side effects include stomach ache, vomiting, diarrhea, or rash. They can apply to either the current antibiotic your child is taking or any prior antibiotic your children have taken.

14) Are you concerned about potential harmful side effects of antibiotics for your child?

- ◯ Yes
- ◯ No
- ◯ I don't know

15) In general, how concerned are you about potential harmful side effects of antibiotics?

- ◯ Slightly concerned
- ◯ Moderately concerned
- ◯ Very concerned

(Read options above.)

16) What side effects of antibiotics are you worried about?

____

17) Has this changed how you give the antibiotic to your child? If does not change anything, you can say that.

____

18) Do you think most of your close friends are concerned about potential harmful side effects of antibiotics for their children?

- ◯ Yes
- ◯ No
- ◯ I don't know

Part III: Counseling from providers/pharmacists

Next, we are going to ask you a few questions about instructions your provider or pharmacist may have given you about your child's antibiotic.

19) When [child's name] was not feeling well and you were thinking about taking him/her to the clinic, did you expect to receive an antibiotic from your doctor?
- ◯ Yes
- ◯ No

20) Why did you expect to receive an antibiotic?

____

21) Did someone at your child's clinic or pharmacy tell you that your child was receiving an antibiotic?
- ◯ Yes
- ◯ No
- ◯ I don't remember

22) Did someone at your child's clinic or pharmacy provide you with instructions for how long to give the antibiotic?
- ◯ Yes
- ◯ No
- ◯ I don't remember

23) Who of the following provided you instructions about how much antibiotic to give your child with each dose?
- ☐ Doctor, Nurse Practitioner, or Physician's Assistant
- ☐ Nurse
- ☐ Pharmacist
- ☐ No one. I read the instructions on the bottle.
- ☐ I don't know or I don't remember

(Read options above and check all that apply.)

24) How long did your child's provider recommend that he/she take the antibiotic? I am going to read some options and you can select one of them.
- ☐ Until the antibiotic runs out
- ☐ Until your child feels better
- ☐ For a specific number of days
- ☐ Your provider did not say
- ☐ Other

25) How long did your child's provider recommend that he/she take the antibiotic?

____

26) Sometimes, there is some antibiotic left in the bottle after the treatment is completed. Did you receive information about how to dispose of any left-over antibiotic?
- ◯ Yes
- ◯ No

27) What instructions were provided about where to dispose of any left-over antibiotic?

____

(If caregiver needs elaborations, instructions could have been verbal from provider or pharmacist or written.)

28) During the clinic visit, were there any questions about the antibiotic you asked or wanted to ask your child's doctor or pharmacist?
- ◯ Yes
- ◯ No

29) What questions about the antibiotic did you ask or want to ask your child's doctor or pharmacist?

____
(Specify which questions caregiver wanted to ask their child's doctor and which ones they wanted to ask their pharamcist.)

30) Did your child's doctor or pharmacist clearly answer your questions about the prescribed antibiotic?

- ◯ Yes
- ◯ No

31) Which questions about antibiotics did your child's doctor or pharmacist not clearly answer?

____
(If the caregiver struggles to answer this question, ask: is there any information you wish had been clarified during your visits?)

Part IV: Dosing and administration

The next portion of the survey is about how you administer the antibiotic to your child.

32) Which tool are you using to administer the antibiotic to your child?

- ◯ Medicine cup
- ◯ Syringe
- ◯ Measuring spoon
- ◯ Tablespoon
- ◯ Other

33) Which tool are you using to administer the antibiotic to your child?

____

34) Sometimes it can be easy to forget to give a child all of the doses of the antibiotic. How many times do you think you have forgotten or missed giving a dose during this antibiotic course?

- ◯ none
- ◯ about 1 - 2 times
- ◯ about 3 - 4 time
- ◯ >4 times
- ◯ do not remember

Part V: Prior antibiotic handling

The following few questions are about how to typically have used antibiotics in the past, including the for current illness of your child. Remember, there are no right or wrong answers and your answers are to help us learn.

35) In the past, when your child/one of your children has been prescribed antibiotics, when did you typically stop giving the antibiotic to your child?

____
(Enter free text capturing caregiver's response. But if caregiver has trouble answering the question, can prompt with "for example, when the prescription date ends, when your child feels better, when the antibiotic runs out, and/or when your child no longer tolerates the antibiotic.")

We have a few additional questions about left-over antibiotics. Sometimes, you have antibiotics left in the bottle after you finish using it for your child.

36) Have you ever had left-over antibiotics from your child/children's illness in your house?

- ◯ Yes
- ◯ No
- ◯ I don't know

(If caregiver asks, clarify that you mean under any scenario.)

37) According to conversations or communications with your friends or relatives, do you think most of your close friends or relatives have ever had left-over antibiotics from their child/children's disease in their homes?

- ◯ Yes
- ◯ No
- ◯ I don't know

38) What have you done with those left-over antibiotics? I'm going to give you some examples of what some people could do. After each example, say "yes" if you have done this or "no" if you have not. You can answer "yes" to more than one option.

- ☐ Keep the antibiotic at home
- ☐ Dispose of the antibiotic
- ☐ Save the antibiotic in case your child is sick again
- ☐ Save the antibiotic in case someone other than your child becomes sick
- ☐ Give or hand-over the antibiotic to someone other than your child who got the prescription

(Check all responses to which caregiver says "Yes.")

39) To whom have you given left-over antibiotics?

- ☐ Your child
- ☐ Someone other than your child
- ☐ Pets or household animals

(Check all that apply. If the parents need prompting, read the individual answer choices below and check if applicable.)

40) Have you given left-over antibiotics from your child/children's illnesses to adults, children, or both?

- ◯ Adults
- ◯ Children
- ◯ Both

41) Do you think most of your close friends or relatives give or hand-over left-over antibiotics to other people?

- ◯ Yes
- ◯ No
- ◯ I don't know

42) To whom do you think your close friends or relatives give left over antibiotics?

- ☐ Their child
- ☐ Someone other than their child
- ☐ Pets or household animals

(Check all that apply. If the parents need prompting, read the individual answer choices above and check if applicable.)

43) Do your close friends or relative given left-over antibiotics to adults, children, or both?

- ◯ Adults
- ◯ Children
- ◯ Both

Part VI: Storage of Antibiotics

The next portion of the survey is about how you store antibiotics. Remember, there are no right or wrong answers and your answers are to help us learn.

44) You mentioned earlier that you have kept left-over antibiotics. Why do you keep left-over antibiotics?

- ☐ Prescriptions are costly.
- ☐ It is inconvenient to go to the doctor's office when my child is sick.
- ☐ You keep them in case your child needs antibiotics again.
- ☐ You keep them in case my doctor will not prescribe antibiotics the next time your child needs them.
- ☐ You keep them in case another one of you family members or friends becomes sick.
- ☐ You just forgot what you had them, but you did not intend to keep them.
- ☐ You weren't sure what to do with the antibiotic
- ☐ Other

(Read answers if need prompting. Check all that apply.)

---

45) Why do you keep left-over antibiotics?

____

---

46) How long do you typically keep antibiotics? If you have not stored antibiotics in the past, how long do you think you will keep the current antibiotic if you have any left-over?

____

(If caregiver requires prompting, state "for example, until the prescription is expired, until the next time a household member develops cold-like symptoms, less than 1 month after a prescription is completed, 1 - 12 months after a prescription is completed, or longer than 12 months.)

---

47) Do you plan to discard left-over antibiotics?

- ◯ Yes
- ◯ No

---

48) How do you plan to discard left over antibiotics?

____

(If caregiver requires prompting, state "for example, do you flush them down the toilet, pour them down the drain of a sink, dispense them in the trash in a bottle or container, dispense them in the trash directly, or return them to the pharmacy?")

Thank you so much for taking the time to answer our questions. Your thoughts about antibiotics are very valuable to us and will help us learn how to improve antibiotic prescription instructions for children.

49) Is there anything else about antibiotics we didn't ask but you would like to tell us?

____

Part VII: Demographic Questionnaire

For the final portion of the survey, we would appreciate if you can take a couple more minutes to answer the following questions about your background. If you do not feel comfortable answering any of these questions, that is ok, and we can skip those questions.

50) What is your age?

____

51) What is your gender?

____

52) Which one or more of the following would you say is your race?

- ☐ White
- ☐ Black or African American
- ☐ Hispanic, Latino/a, or Spanish origin
- ☐ American Indian or Alaska Native
- ☐ Asian/Pacific Islander
- ☐ Not sure
- ☐ Prefer not to answer

(Read options above and check all that apply.)

53) Are you __________ ?

- ◯ Married
- ◯ In a domestic partnership
- ◯ Single
- ◯ Prefer not to answer

(Read options above.)

54) How many children do you have?

____

55) What is the highest grade or year of school you completed?

- ◯ Never attended school or only attended kindergarten
- ◯ Grades 1 - 8 (elementary school)
- ◯ Grades 9 - 11 (some high school)
- ◯ Grade 12 or GED (high school graduate)
- ◯ College 1 year to 3 years
- ◯ College 4 years or more (college graduate)
- ◯ Graduate degree (masters or doctorate)
- ◯ Prefer not to answer

(Read options above.)

56) What is your annual household income from all sources?

- ◯ $100,000
- ◯ Prefer not to answer

(Read options above.)

Again, all of your responses to our questions will be anonymous in our study records. Before we go, we would like to measure the amount of left-over antibiotic and swab your child's nose one last time. After completing these steps, as a thank you for your time and participation, we would like to give you this $35 gift card to Walmart. Before we are able to give this to you, we need to record the following information for tax purposes:

57) Name of caregiver or child: __________________________________

58) SSN of caregiver or child listed above: __________________________________

59) Home address: __________________________________

60) Can we please measure the amount of left-over antibiotics?
- ◯ Yes
- ◯ No

(If caregiver responds yes, measure left over antibiotic and record amount in question 51. First, pour antibiotics into container. Then, use syringe to measure antibiotic as you pour antibiotic back into original bottle.)

61) Amount of antibiotic prescribed (mL): __________________________________
(Calculate amount of antibiotic prescribed in mL.)

62) Amount of antibiotic dispensed (mL): __________________________________

63) Amount of left over antibiotic (mL): __________________________________

64) Now, we will swab your child's nose before we go. (Was the swab collected?)
- ◯ Yes
- ◯ No

65) Post-antibiotic NP specimen number: __________________________________

66) Gift card tracking number: __________________________________
(Record gift card number, hand it to caregiver, thank him/her and say goodbye before leaving.)
