# Supplementary material for: Antibiotic perceptions, adherence, and disposal practices among parents of pediatric patients
Source: PLoS One. 2023 Feb 9;18(2):e0281660. doi: 10.1371/journal.pone.0281660 (PMC9910628; doi:10.1371/journal.pone.0281660)
Supplement: S1 Table — We report key quotes to illustrate each component of the framework. Each quote identifies the participant number, antibiotic prescribed, and child’s ARI diagnosis. (DOCX) [file pone.0281660.s002.docx]

| **Clinic Visit** | |
| --- | --- |
| **Antibiotic beliefs** | |
| Complete understanding | Quote 1 (Q1). “I think it's a good medicine that helps fight off extra bacteria that sometimes us parents can't determine.” (Participant 1, Amoxicillin, Acute Otitis Media) |
|  | Q2. “I think it fights the bad bacteria.” (Participant 9, Amoxicillin, Acute Otitis Media) |
| Incomplete understanding | Q3. “Yeah, like something that boosts your immune system to fight off whatever needs to be.” (Participant 13, Amoxicillin, Streptococcal Pharyngitis) |
|  | Q4. “It helps with possibly the white blood cells, but I'm not sure.” (Participant 6, Amoxicillin, Acute Otitis Media) |
|  | Q5. “A cure to just all type of just bacteria and infections.” (Participant 6, Amoxicillin, Acute Otitis Media) |
| Complete misunderstanding | Q6. “I'm guessing it helped by reducing whatever pain that was in her ear.” (Participant 3, Amoxicillin, Acute Otitis Media) |
|  | Q7. “I think it just creates the antibodies in the body to fight off the infection is my understanding of how they work." (Participant 13, Amoxicillin, Streptococcal Pharyngitis) |
|  | Q8. “I mean, they put them in the IV, so I would just think that they go through the blood stream. It helps prevent stuff.” (Participant 2, Amoxicillin, Acute Otitis Media) |
|  | Q9. “I guess it's designed to tackle whatever is going on. It's specifically made to attack. If you had a cough, it's designed to protect the cough or if he had a fever.” (Participant 3, Amoxicillin, Acute Otitis Media) |
| **Antibiotic Expectations** | |
| Antibiotics work well | Q10. “If you look up antibiotics or just a certain antibiotic you have so many cures for that one type of medication that people don't realize. Like one medication can help a lot of things." (Participant 11, Amoxicillin, Acute Otitis Media) |
|  | Q11. “Well, they just say it doesn't help or cure and I'm like, y'all would be amazed what antibiotics do. Her cough is gone.” (Participant 11, Amoxicillin, Acute Otitis Media) |
|  | Q12. “But for the most part, I am pro-antibiotic because I know it's going to help with the immune system as best as possible.” (Participant 6, Amoxicillin, Acute Otitis Media) |
| Previous experience with antibiotics | Q13. “Because she had the bite on her arm and her eye and it just helped, I guess, the pain and the swelling go down to the regular skin type.” (Participant 4, Amoxicillin & Clindamycin, Preseptal Cellulitis) |
| Antibiotics did not meet expectations | Q14. “ … from my experience, usually the medicine, they just give you an antibiotic, starts working pretty quickly. Then that's why I was thinking by the second day that he was getting worse, and I knew in my own experience that always seems like you get quite a bit better, pretty quick." (Participant 13, Amoxicillin, Streptococcal Pharyngitis) |
| Experience with side effects from antibiotics | Q15. “ … when I gave him that, it ended up he had flared up with a rash and so I had to take him back to the doctor. She's saying because they switched his antibiotic and taking the antibiotic before and not recently, and they was like not long for him to take another different kind of antibiotic.” (Participant 12, Amoxicillin, Acute Otitis Media) |
|  | Q16. “No, but I will say that I did notice a small rash yesterday and it was like, ‘hm, what is that?’ And now that you're saying that I think that they may have just been a side effect of the medicine.” (Participant 5, Clindamycin, Preseptal Cellulitis) |
|  | Q17. “ … I used to work with infants, and all the infants that I would see that came in on amoxicillin would have dark stool or diarrhea or something like that. So, I was worried that I was going to see some of those side effects with them.” (Participant 8, Amoxicillin, Acute Otitis Media) |
| Need for basic information on how antibiotics work | Q18. “I just wish that the doctor would have explained how exactly it works in the body. Because I don't really know, I mean I know what an antibiotic does obviously, but like I don't know how this specific one helps with an ear infection. Does it prevent infections, or does it fight off the infection it already has, just how does it work?” (Participant 8, Amoxicillin, Acute Otitis Media) |
| **Instructions received during clinic visit** | |
| Source of instructions | Q19. “Yes, her doctor.” (Participant 4, Amoxicillin & Clindamycin, Preseptal Cellulitis) |
|  | Q20. “The lady that checked him out told me before the end of the visit that he'd be on antibiotic.” (Participant 5, Clindamycin, Eye infection) |
|  | Q21. “Both the doctor and the pharmacist. (Participant 8, Amoxicillin, Acute Otitis Media) |
| Type of antibiotic | Q22. “I believe it was amoxicillin.” (Participant 3, Amoxicillin, Acute Otitis Media) |
|  | Q23. “It was the amoxicillin and then cin... it starts with a C”. (Participant 4, Amoxicillin & Clindamycin, Preseptal Cellulitis) |
|  | Q24. [asked what medications were given] “Just the antibiotic.” (Participant 6, Amoxicillin, Acute Otitis Media) |
|  | Q25. “It was amoxicillin and... It's Tylenol, but it's another name for it and it starts with an A.” (Participant 2, Amoxicillin, Acute Otitis Media) |
| Instructions for use | Q26. “The specific number of days. For 10 days." (Participant 13, Amoxicillin, Streptococcal pharyngitis) |
|  | Q27. “Two doses, 10 days.” (Participant 6, Amoxicillin, Acute Otitis media) |
|  | Q28. “Yeah, and it was seven milligrams three times a day and I'm like "that's a lot". But it worked. It definitely worked.” (Participant 5, Clindamycin, Preseptal Cellulitis) |
|  | Q29. “Until it's all gone.” (Participant 3, Amoxicillin, Acute Otitis Media) |
| Disposal Instructions | Q30. “The doctor said I could throw it away or just give him the rest, the same dosage.” (Participant 8, Amoxicillin, Acute Otitis Media) |
|  | Q31. “The pharmacist told us when you picked it up, that we could just throw it in the trash.” (Participant 10, Amoxicillin, Acute Otitis Media) |
|  | Q32. “I don't think I did either. I was told he will have leftovers, but I don't think I was told specifically.” (Participant 13, Amoxicillin, Streptococcal Pharyngitis) |
|  | Q33. “They just said to get rid of it if I wanted to.” (Participant 4, Amoxicillin & Clindamycin, Preseptal Cellulitis) |
|  | Q34. “So, I mean they never tell me to like throw it out. So, I don't feel like it's wrong. I don't know.” (Participant 2, Amoxicillin, Acute Otitis Media) |
| **Treatment and Adherence** | |
| **Daily Adherence** | |
| Inconvenient timing | Q35. “… I only missed one and that was yesterday because it was 2:30 in the morning, and I had the alarm set for 1:30 and I was like ‘okay, I'll just get up in an hour’ and did not get up in an hour. I don't know why I didn't just reset the alarm.” (Participant 5, Clindamycin, Preseptal Cellulitis) |
| Forgetting | Q36. “We just missed the first one a day, because I just didn't give it to her.” (Participant 10, Amoxicillin, Acute Otitis Media) |
| Busy schedule | Q37. “Only because I'm busy a lot so it's like I don't... I try to give it to her in the morning, and then I tried to get my mom to give it to her when she picks her up from daycare, or something like that. But sometimes I do forget it.” (Participant 2, Amoxicillin, Acute Otitis Media) |
|  | Q38. “Either I'm rushing to get to work or tired from …” (Participant 11, Amoxicillin, Acute Otitis Media) |
| **Stopping** | |
| When the medication runs out | Q39. “For myself, yeah. I usually just taking it until it's all gone. Yeah, like when you have a really bad cold and I just finish it.” (Participant 8, Amoxicillin, Acute Otitis Media) |
|  | Q40. “Well, it was done when I was finished. The last draw was the last of what was in the bottle. So when I was done with that, I just threw the bottle out and that was that.” (Participant 5, Clindamycin, Preseptal Cellulitis) |
| Specific number of days | Q41. “Just the specific number of days.” (Participant 9, Amoxicillin, Acute Otitis Media) |
| When child is feeling better | Q42. “But after maybe a certain time I stopped giving it to him because it came out by itself, so I just threw it away when I didn't need it anymore.” (Participant 7, Amoxicillin, Acute Otitis Media) |
|  | Q43. “When they felt better.” (Participant 2, Amoxicillin, Acute Otitis Media) |
|  | Q44. “Yeah, basically until the symptoms clear up or whatever.” (Participant 6, unclear, Acute Otitis Media) |
|  | Q45. “Depends on what the infection is. I usually give about a bottle or more then stop.” (Participant 11, Amoxicillin, Acute Otitis Media) |
| **Disposal** | |
| **Keep leftovers** | |
| Cost | Q46. “Yes, that too, because her antibiotics were $30.” (Participant 9, Amoxicillin, Acute Otitis Media) |
| Give to someone else | Q47. “I'm not going to lie. My granny have, my auntie have gave it to me or each other.” (Participant 11, Amoxicillin, Acute Otitis Media) |
|  | Q48. “I wouldn't give it to my daughter, but I would give it to another adult for another thing. (Participant 7, Amoxicillin, Acute Otitis Media)  Q49. “Well, I don't know if it was antibiotic, but sometimes when we have medicine, and since there are so many kids here, or even if some of us adults get sick, if they know what type it is, we keep it, and then if somebody's sick we'd let them take it. Like, if my grandma is sick or something like that, she don't live here, but if I have it I'll give it. So, I mean they never tell me to like throw it out. So, I don't feel like it's wrong. I don't know.” (Participant 2, Amoxicillin, Acute Otitis Media) |
|  | Q50. “I use it until the expiration date and then I'll throw it away. (Participant 9, Amoxicillin, Acute Otitis Media) |
| Save for the same child | Q51. “Yeah, like to their child again, or another child in the household, or like obviously if we can take it. You know, as long as it's those baby doses, then we can take it.” (Participant 2, Amoxicillin, Acute Otitis Media) |
| **Disposal** | |
| Trash | Q52. “No, if there's some left, I just probably throw it away or something like that.” (Participant 12, Amoxicillin, Acute Otitis Media) |
| Drain | Q53. “Probably just throw it in the trash or pour it down the sink and then put it in the trash.” (Participant 6, Amoxicillin, Acute Otitis Media) |
|  | Q54. “Since it's a legal output, I would probably just pour it in the sink and rinse the sink out and throw all the bottles in the trash.” (Participant 5, Clindamycin, Preseptal Cellulitis)  Q55. “Poured out in a drain and take off the label.” (Participant 3, Amoxicillin, Acute Otitis Media) |
|  | Q56. “[…] I think you can pour it down the toilet, right? (Participant 13, Amoxicillin, Streptococcal pharyngitis) |
|  | Q57. “Yeah, no I've disposed of a... I don't think I've ever had any left a random box of my own. But I've taken old medicine to the disposal at pharmacies or different drop off locations before.” (Participant 13, Amoxicillin, Streptococcal Pharyngitis) |
| Unclear | Q58. “He didn't say how to dispose of it. He just told me that I should, he basically just said throw it away because it's a liquid, it's in a bottle, and he just said throw it away if nobody's going to use it.” (Participant 5, Clindamycin, Preseptal Cellulitis) |
