# Supplementary material for: Antibiotic perceptions, adherence, and disposal practices among parents of pediatric patients
Source: PLoS One. 2023 Feb 9;18(2):e0281660. doi: 10.1371/journal.pone.0281660 (PMC9910628; doi:10.1371/journal.pone.0281660)
Supplement: S2 Table — (DOCX) [file pone.0281660.s003.docx]

| **Question** | **Yes**  **N (%)**  **(N = 13)** |
| --- | --- |
| **General** | |
| Do you think the antibiotic helped your child get over his/her illness? | 12 (92) |
| **Side effects** | |
| Are you concerned about potential harmful side effects for your child? | 6 (46) |
| Do you think most of your close friends are concerned about potential harmful side effects of antibiotics for their children? | 2 (15) |
| **Counseling from providers/pharmacists** | |
| Did you expect to receive an antibiotic from your doctor? | 7 (47%) |
| Did someone at your child’s clinic or pharmacy tell you that your child was receiving an antibiotic? | 12 (92) |
| Did someone at your child’s clinic or pharmacy provide you with instructions for how long to give the antibiotic? | 12 (92) |
| Did you receive information about how to dispose of any leftover antibiotic? | 8 (62) |
| **Disposal of antibiotics** | |
| Have you ever had left-over antibiotics from your child/children’s illness in your house? | 8 (62) |
| Do you think most of your close friends or relatives have had leftover antibiotics from their children’s illnesses in their homes? | 13 (100) |
| Do you think most of your close friends or relatives give or hand over leftover antibiotics to other people? | 6 (46) |
| Do you plan to discard leftover antibiotics? | 11 (85) |
| What have you done with leftover antibiotics? | |
| Give or hand over the antibiotic to someone other than the child. | 2 (15) |
| Save the antibiotic in case someone other than the child becomes sick. | 3 (23) |
| Save the antibiotic in case the child is sick again. | 5 (38) |
| Dispose of the antibiotic. | 7 (54) |
| Keep the antibiotic at home. | 7 (54) |
| Why do you keep left-over antibiotics? | |
| You weren’t sure what to do. | 4 (31) |
| You forgot you had them. | 6 (46) |
| In case family or friends become sick. | 4 (31) |
| In case the doctor won’t prescribe antibiotics. | 1 (8) |
| In case the child needs antibiotics again. | 5 (38) |
| It’s inconvenient to go to the doctor. | 3 (23) |
| Prescriptions are costly. | 2 (15) |
