# Supplementary material for: Antibiotic perceptions, adherence, and disposal practices among parents of pediatric patients
Source: PLoS One. 2023 Feb 9;18(2):e0281660. doi: 10.1371/journal.pone.0281660 (PMC9910628; doi:10.1371/journal.pone.0281660)
Supplement: Attachment — Submitted filename: Article review -PLOS.docx [file pone.0281660.s004.docx]

**Article review -PLOS**

1. Recommendation -Minor Revision
2. Is the manuscript technically sound, and do the data support the conclusions?

Answer Yes

3. Has the statistical analysis been performed appropriately and rigorously?

Answer Yes

4. Does the manuscript adhere to the PLOS Data Policy? Answer: Yes

5. Is the manuscript presented in an intelligible fashion and written in standard English? Answer Yes
6. Review Comments to the Author (Minimum 200 characters)

Manuscript entitled: "Antibiotic perceptions, adherence, and disposal practices among parents of pediatric patients: a mixed-methods study."

**Good points**

1.    The introduction is appropriate.

2.    The Inclusion and Exclusion criteria have been appropriately included.

3.    Pilot testing was done and consent was taken .Semi structured interviews with audio recording was a good idea.

4.    Quantitative data was transcribed by the professionals and this was again a good idea.

**Please explain and make amends accordingly**

1. Why was a gift coupon worth $35, given to the participants? This seems to be unethical.

2. Sample size seems to be too small. How did you determine the sample size and what were your limitations?

3. Please mention what additional information you could derive from the structured interviews, besides that obtained from the questionnaire. Was data saturation taken into consideration when piloting the questionnaire? Did questionnaire planning precede the interview?

4. Reference 11, Page number missing.

5. What was the need for the team members to meet when decoding the interview transcripts? Could it have given rise to some bias and how did you take care of the investigators bias.

**Other Points:**

1. Title page missing

2. Table caption can be added.

3. Funding acknowledgement not to be done anywhere in the manuscript .Please check if salary support is a funding acknowledgement.

7. Would you like your identity revealed to the authors of this submission?

Answer: No
8. Do you have any potentially competing interests?

Answer: "None."

9. Do you want to get recognition for this review on Publons

Answer: No
